# Perforation of the Papilla of Vater in Wire-Guided Cannulation

**DOI:** 10.1155/2016/5825230

**Published:** 2016-06-05

**Authors:** Yuichi Takano, Masatsugu Nagahama, Eiichi Yamamura, Naotaka Maruoka, Hiroshi Takahashi

**Affiliations:** Division of Gastroenterology, Department of Internal Medicine, Showa University Fujigaoka Hospital, Yokohama, Kanagawa 227-8501, Japan

## Abstract

*Background.* WGC in ERCP is considered a safe technique, although rare complications can occur. One unique complication of WGC is the perforation of the papilla of Vater by the guidewire.* Subjects and Methods.* Of 2032 patients who underwent ERCP at our department between January 2010 and December 2014, we selected 208 patients who underwent WGC for naïve papilla as subjects. A detailed examination of patients in whom a perforation occurred was conducted, and risk factors for perforations were investigated.* Results.* The perforation was observed in 7 of 208 patients (3.4%). All patients recovered with conservative treatment without the need for surgery. The perforation rate was significantly higher in the patients with juxtapapillary duodenal diverticula than those without diverticula (12.5% versus 0.6%, *p* < 0.001). Cannulation of the bile duct was ultimately achieved in 5 of 7 patients; PSP was performed for 4 of these patients.* Conclusion.* Caution must be exercised when dealing with patients who have a juxtapapillary duodenal diverticula because they are at higher risk of perforations. Because these are small perforations made by a wire, most of them heal with conservative treatment. However, perforations can make cannulation difficult, and PSP may be useful for deep cannulation.

## 1. Introduction

Endoscopic retrograde cholangiopancreatography (ERCP) is a modality that is indispensable in the diagnosis and treatment of pancreaticobiliary diseases. In recent years, there have been remarkable improvements in techniques and devices for ERCP. Wire-guided cannulation (WGC) has become widely adopted mainly in Europe and the United States for deep cannulation. WGC is a method of deep cannulation in which a guidewire is loaded into a catheter (C) or a sphincterotome (S) and is advanced into the bile duct. It is considered safe, but rare complications do occur. One unique complication of WGC is the perforation of the papilla of Vater by the guidewire. To date, very few studies have focused on the perforation of the papilla of Vater in WGC.

## 2. Subjects and Methods

We conducted a retrospective cohort study. Of 2032 patients who underwent ERCP at our department between January 2010 and December 2014, we selected 208 patients who underwent WGC for naïve papilla as subjects (116 men and 92 women; mean age: 70.1 ± 10.9 years). One hundred and twelve patients had benign biliary tract disease (choledocholithiasis in 88, acute cholangitis in 12, benign bile duct stenosis in 9, bile leakage in 2, and obstructive jaundice in 1). A total of 36 patients had malignant biliary tract disease (extrahepatic bile duct carcinoma in 23, intrahepatic cholangiocellular carcinoma in 5, gallbladder cancer in 7, and hepatic metastasis from lung cancer in 1), 10 had benign pancreatic disease (chronic pancreatitis in 9, intraductal papillary mucinous neoplasm in 1), 39 had malignant pancreatic disease (pancreatic cancer in 38 and pancreatic metastasis from lung cancer in 1), and 11 had cancer of the papilla of Vater.

The papilla of Vater was untreated in all patients. One hundred and sixty patients did not have juxtapapillary duodenal diverticula, while 48 did. Two hundred and one patients did not have a history of gastric surgery, whereas 7 did (Billroth I in 1, Billroth II in 2, and Roux-en-Y in 4).

Duodenoscopes with a backward viewing angle of 15° (JF-260V, Olympus Medical Systems Corp, Tokyo, Japan) and single balloon endoscopy (SIF-Q260, Olympus) were used in this study. The catheters used were PR-104Q, PR-128Q, and PR-V614M (Olympus). The guidewires used were a 0.035-inch straight jagwire (Boston Scientific Japan, Tokyo, Japan) and a 0.025-inch angle first-generation visiglide (Olympus). The sphincterotome used was a CleverCut3V (Olympus). The assistant (experienced physician; ERCP experience over 500 cases) manipulated the guidewire.

**Figure 1:**
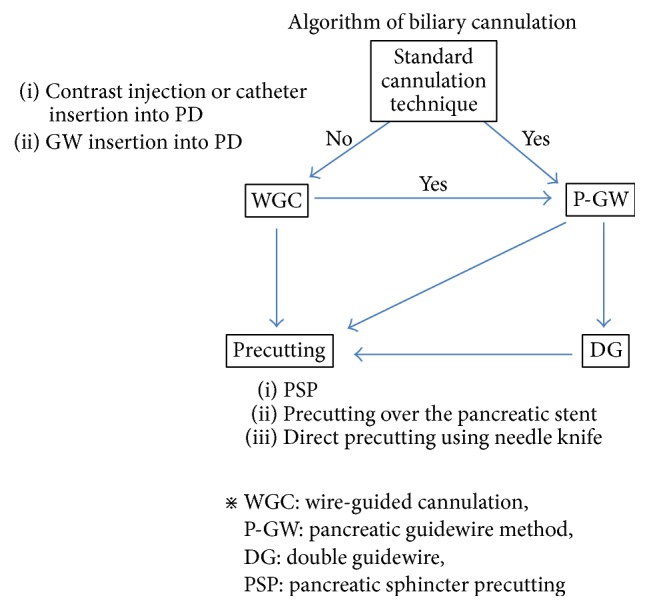
Our algorithm for biliary cannulation is shown. At our hospital, cannulation with a catheter is typically the first choice, and WGC is performed only for difficult cases. If the deep cannulation could not be achieved with standard approach within 10 minutes, WGC is performed.

Our algorithm for biliary cannulation is shown in Figure 1. At our hospital, cannulation with a catheter is typically the first choice, and WGC is performed only for difficult cases. If the deep cannulation could not be achieved with standard approach within 10 minutes, WGC is performed. Therefore, in this study, WGC is not used in the narrow sense of never using a contrast medium but is rather used in a broader sense that WGC includes so-called wire-loaded cannulation when contrast is possible but deep cannulation is not.

The definition of the perforation of the papilla of Vater used in this study is “intraluminal or extraluminal perforation by the guidewire that is observed on the endoscope or fluoroscope screen.” Perforations were classified as intraluminal or extraluminal.

Cases of perforations were examined in detail, and the risk factors for perforations were investigated. Chi-square for independence test was used for statistical analysis.

## 3. Results

**Figure 2:**
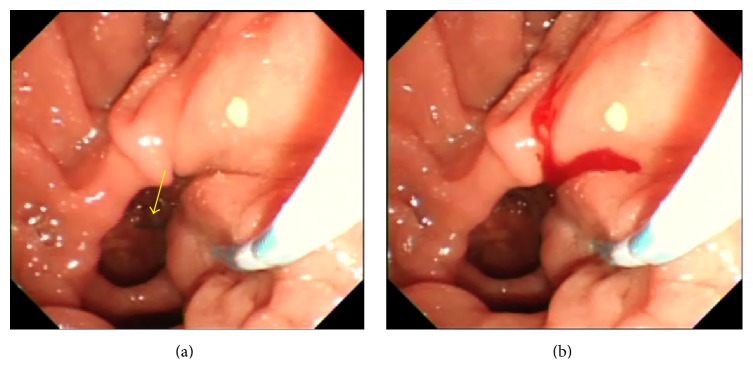
(a) 74-year-old woman who had juxtapapillary duodenal diverticula underwent ERCP for choledocholithiasis. WGC (C + 0.035-inch straight jagwire) was attempted; however, an intradiverticular perforation by the wire was observed on the endoscope screen (arrow). (b) A small amount of bleeding was observed when the perforation occurred. WGC was repeated, but the guidewire passed through the false lumen created by the perforation, and thus biliary cannulation was not possible. Biliary cannulation was achieved by retracting the scope and applying torque to the left.

**Figure 3:**
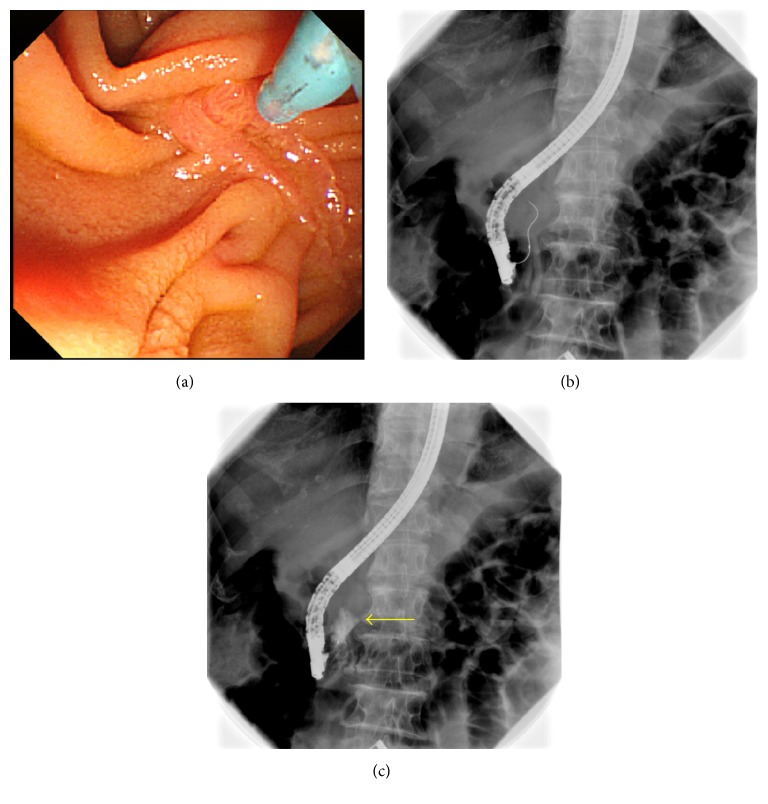
(a) An 80-year-old man with no juxtapapillary duodenal diverticula underwent ERCP for obstructive jaundice that was induced by cancer of the head of the pancreas. Because of the invasion of pancreatic head cancer to duodenum, having a front view of a papilla of Vater is difficult. (b, c) WGC (S + 0.025-inch angle visiglide) was performed, but the wire made an extraluminal perforation, and extravasation of the contrast medium was observed during fluoroscopy (arrow). After PSP was performed with a pancreatic duct guidewire, deep cannulation of the bile duct and biliary drainage were achieved.

The success rate of WGC was 86.6% (179/208) and the overall success rate of biliary cannulation was 98.1% (204/208) in this study. A perforation of the papilla of Vater was observed in 7 of 208 patients (3.4%) who underwent WGC. Of these, 4 had intraluminal (intradiverticular) perforations, and 3 had extraluminal perforations (Figures 2 and 3). All patients recovered with conservative treatment without the need for surgery. Prior to changing to WGC, cholangiography was possible in 3 patients but was not possible in the remaining 4 patients. Cases in which a perforation was observed are listed in Table 1. The mean age of 7 patients with perforation was 77 years (65–88), of whom 5 were men and 2 were women. Six had juxtapapillary duodenal diverticula and 1 did not. A 0.035-inch jagwire was used for 4 patients, and 0.025-inch visiglide was used for the other 3. There were no perforations by the cannula itself.

The rates of perforation of the papilla of Vater in each factor were also investigated (Table 2). The perforation rate was significantly higher in the patients with juxtapapillary duodenal diverticula than those without diverticula (12.5% versus 0.6%, *p* < 0.001). No difference in the rate of perforations was observed between 0.035-inch guidewire and 0.025-inch guidewire (3.6% versus 3.1%).

Biliary cannulation was achieved in 5 of 7 patients, and pancreatic sphincter precutting (PSP) was performed for 4 of those patients. Of 2 patients for whom cannulation was not possible, 1 underwent elective surgery (choledocholithotomy), and the other recovered with conservative treatment. Computed tomography scans were taken after 1-2 days in patients with extraluminal perforations, but these revealed no findings such as free air or extravasation of the contrast medium in any patient.

## 4. Discussion

WGC has come to be widely adopted primarily in Europe and the United States as a cannulation technique for ERCP. This is because several randomized controlled trials and meta-analysis have shown that WGC achieves a higher cannulation rate than normal cannulation methods and leads to a lower incidence of pancreatitis [1–7]. The reason why WGC reduces the incidence of pancreatitis is that pancreatography, which is considered a risk factor for post-ERCP pancreatitis, is avoided [1–7]. However, other randomized controlled trials have also shown that the incidence of pancreatitis and the rate of cannulation do not differ from those of normal cannulation methods [8–10]; therefore, a consensus has not yet been reached. WGC is considered a safe method, but rare complications can occur. One unique complication is the perforation of the papilla of Vater by the guidewire. Complications of portal vein cannulation with WGC have also been reported [11, 12].

Kawakami et al. [10] defined perforations of the ampulla of Vater as perforations caused by the guidewire passing through the posterior wall of the ampulla of Vater, and they report that these perforations occurred in 2% (4/199) of the patients they studied. They also noted that these perforations were minor and resolved without therapy. Adler et al. [13] defined the guidewire perforation as “guidewire passage out of the duodenum but not into either the biliary or pancreatic ducts” and noted that it occurred in 1.3% (11/822) of the patients they studied. Deep cannulation was eventually achieved in 10 of these patients (91%), and all patients recovered with conservative treatment. Mohammad Alizadeh et al. reported an incidence of perforations of 0.9% (1/546), but they did not describe the cases in detail, and thus it is unclear whether these perforations were caused by a guidewire or a scope [14]. On the other hand, many studies reported an incidence of perforations in WGC of 0% [1–5, 8, 9, 15]. However, they did not clearly define perforations, and their definitions may not have included perforations caused by a guidewire. In summary, these studies indicate that the perforation of the papilla of Vater occurs in WGC in approximately 0%–2% of cases.

Perforations of the papilla of Vater caused by a wire must be considered separately from those caused by other implements. They are very small perforations; thus, most of them can be treated conservatively. All of our 7 patients recovered with conservative treatment, and surgery was not required.

In a study of 12,427 patients who underwent ERCP, Fatima et al. reported that 75 (0.9%) had perforations [16]. Twenty-four of these perforations were caused by a guidewire, and 88% (21/24) of these patients recovered with conservative treatment. Eight perforations were caused by a scope, and 13% (1 patient) recovered with conservative treatment, whereas 87% (7 patients) required surgery. Surgery was similarly required for 36% (4/11) of perforations caused by EST. In summary, most perforations caused by a guidewire healed with conservative treatment, whereas many perforations caused by a scope or EST required invasive treatment including surgery. In recent years, there have also been reports of the successful treatment of post-EST perforations by placement of a self-expanding metallic stent [17].

Perforations of the papilla of Vater can be classified into 2 categories. The first is intraluminal (intradiverticular) perforations, and the second is extraluminal perforations. Risks of panperitonitis and inflammation of the retroperitoneum are low with intraluminal perforations. When extraluminal perforation occurs, gastrointestinal fluids (including bile and pancreatic juice) may leak outside of the lumen; therefore, the patient must be monitored for panperitonitis and inflammation of the retroperitoneum. When possible, drainage of the perforated bile duct or the pancreatic duct should be performed. However, drainage was only possible for 1 of our 3 patients with extraluminal perforations. Drainage was not possible for the remaining 2 patients, but they recovered with conservative treatment. Such perforations are very small, which is likely why most of them can be treated conservatively.

With both intraluminal and extraluminal perforations, a false lumen is formed, and papillary edema develops. This phenomenon can make cannulation difficult. Four of 5 patients for whom biliary cannulation was finally achieved underwent PSP, indicating that PSP may be useful for deep cannulation. PSP is a method established as a precut technique [18] for patients for whom cannulation is difficult and can open a path from the false lumen to the true bile duct. Some disadvantages of this method include the fact that it cannot be performed when the guidewire cannot be inserted into the pancreatic duct, it cannot be performed for patients who are prone to bleeding, and using this method requires expertise [18].

In our study, we noted a higher rate of the perforation of the papilla of Vater compared with previous studies. The reason for this is likely that we only performed WGC for patients for whom cannulation was difficult. Furthermore, when cannulation was performed with the standard approach for 10 min until WGC, some patients developed papillary edema. Therefore, in such instances, compared to the case when WGC is performed from the beginning, it is possible that perforation tends to occur.

## 5. Conclusion

The perforation of the papilla of Vater is a rare complication associated with WGC that sometimes occurs. Caution must be exercised when dealing with patients who have a juxtapapillary duodenal diverticula because they are at higher risk of perforations. Perforations are classified as intraluminal or extraluminal based on the site of the perforation. Because they are small perforations made by a wire, most of them can be treated conservatively. However, biliary cannulation can be difficult after perforation occurs; thus, different cannulation strategies are required. PSP may be useful for this purpose.

## Figures and Tables

**Table 1:** Details of perforation cases.

	Age	Sex	Indication for ERCP	Duodenal diverticulum	Device	Site of perforation	Deep cannulation	Precut	CT findings
Case 1	76	F	CBD stone	Yes	C + 0.025-inch guidewire	Intraluminal	Success	PSP	Not performed
Case 2	86	M	Obstructive jaundice	Yes	C + 0.025-inch guidewire	Extraluminal	Unsuccess	None	No free air, no extravasation
Case 3	88	M	CBD stone	Yes	C + 0.025-inch guidewire	Extraluminal	Unsuccess	None	No free air, no extravasation
Case 4	80	M	Pancreatic cancer	None	S + 0.025-inch guidewire	Extraluminal	Success	PSP	No free air, no extravasation
Case 5	74	F	CBD stone	Yes	C + 0.035-inch guidewire	Intraluminal	Success	None	Not performed
Case 6	65	M	CBD stone	Yes	C + 0.035-inch guidewire	Intraluminal	Success	PSP	Not performed
Case 7	72	M	CBD stone	Yes	C + 0.025-inch guidewire	Intraluminal	Success	PSP	Not performed

**Table 2:** Perforation rate for each factor.

	Perforation rate	*p* value
Juxtapapillary duodenal diverticulum (−) versus (+)	0.6% (1/160) versus 12.5% (6/48)	<0.001
0.025-inch guidewire versus 0.035-inch guidewire	3.1% (3/98) versus 3.6% (4/110)	n.s.
C + guidewire versus S + guidewire	3.2% (6/188) versus 5% (1/20)	n.s.
Benign disease versus malignant disease	4.9% (6/122) versus 1.2% (1/86)	n.s.

Chi-square for independence test.
